# Effect of the PB2 and M Genes on the Replication of H6 Influenza Virus in Chickens

**DOI:** 10.1155/2014/547839

**Published:** 2014-02-18

**Authors:** Hiroichi Ozaki, Yi Guan, Malik Peiris, Robert Webster, Ayato Takada, Richard Webby

**Affiliations:** ^1^Laboratory of Veterinary Microbiology, Faculty of Agriculture, Tottori University, 4-101 Koyama, Tottori 680-8553, Japan; ^2^Centre for Influenza Research, School of Public Health, The University of Hong Kong, Hong Kong; ^3^Division of Virology, Department of Infectious Diseases, St. Jude Children's Research Hospital, Memphis, TN 38105, USA; ^4^Division of Global Epidemiology, Research Center for Zoonosis Control, Hokkaido University, Sapporo 001-0020, Japan

## Abstract

H6 subtype influenza viruses are commonly isolated from wild aquatic birds. However, limited information is available regarding H6 influenza virus isolated from chickens. We compared the viral genome segment between A/chicken/Hong Kong/W312/97 (H6N1), which was able to grow in chicken trachea, and A/duck/Shantou/5540/01 (H6N2), which was isolated from wild aquatic duck, to explore the factors for effective replication in chicken. When chickens were inoculated with 7 + 1 reassortants (W312 background), the replication of viruses with PB2 and M genes derived from the duck strain was significantly reduced. Chimeras of PB2 and M proteins, encoding the C-terminal region of the PB2 protein and the M2 protein from W312, were required for efficient replication in canine-derived (MDCK) cells and in chicken trachea. These results indicate that host range may be determined by some types of internal proteins such as PB2 and M2, as well as by surface glycoprotein like hemagglutinin.

## 1. Introduction

Avian influenza viruses (AIVs) are member of the family Orthomyxoviridae, genus *Influenzavirus* A [1]. Influenza A viruses are classified according to the antigenic properties of their hemagglutinin (HA) and neuraminidase (NA) surface glycoproteins. Till date, 16 HA subtypes and 9 NA subtypes have been recognized [2, 3]. Moreover, a novel subtype, H17N10, isolated from yellow-shouldered bats was recognized [4]. Influenza viruses are normally nonpathogenic in their natural hosts in which they remain in evolutionary stasis [2, 3].

H6 influenza viruses are frequently isolated from wild aquatic birds and domestic ducks, and most of them are nonpathogenic to avian. They have a broader host range than any other subtype [5, 6]. H6 viruses continue to circulate worldwide including North America and South Africa [6–10], as well as Asian countries including southern China and Taiwan [11, 12]. A/teal/Hong Kong/W312/97 (H6N1) (W312)-like virus has been endemic in southern China since the late 1990s [13, 14]. Limited information is available regarding the frequency of infection of H6 viruses isolated from chickens [9, 12, 15], in contrast to quail and minor domestic poultry [11, 14, 16, 17].

The host range of influenza A virus is determined by amino acid residue substitutions or receptor binding specificity. The HA molecule primarily contributes to the determination of host range by receptor specificity [1]. In contrast, internal viral proteins such as polymerases are required to replicate the viral genome in the host cell.

We previously reported that the H6 isolate from ducks replicates poorly in chicken trachea and that the viruses recovered were W312-like viruses [16]. The aim of this study was to determine the factors required for effective viral replication in different host cells. We generated 7 + 1 reassortant viruses in which one genome segment derived from W312 virus was replaced with that of A/duck/Shantou/5540/01 (H6N2) (ST) isolated from wild aquatic birds and verified the viral factors required for virus replication in cultured cells and chicken trachea.

## 2. Materials and Methods

### 2.1. Viruses, Cells, and Plasmids

A/teal/Hong Kong/W312/97 (H6N1) and A/duck/Shantou/5540/01 (H6N2) were propagated in 10-day-old chicken eggs. The Madin-Darby canine kidney (MDCK) cell line was maintained in minimal essential medium (MEM; Life Technologies) containing 10% fetal bovine serum.

Full-length cDNA copies of viral genes were amplified using the reverse-transcriptase- (RT-) polymerase chain reaction (PCR) with previously described primers [18]. The plasmids carrying the eight gene segments of the W312 and ST strains were constructed following this method. Briefly, each PCR fragment was digested with *Bsa*I (for PB2 and NA genes) or *Bsm*BI (the other six genes) and ligated to vector DNA (pHW2000) [19]. We determined the full-length nucleotide sequences of all clones. Chimeric PB2 genes were constructed by exchanging the two fragments generated from the *Afl*II-digestion fragments of the W312 and ST-PB2 genes (WST-PB2 *Afl*II and STW-PB2 *Afl*II). Chimeric M genes were designed to express W312-M1 and ST-M2 proteins (WM1-STM2) or ST-M1 and W312-M2 proteins (STM1-WM2). Each chimeric M gene was constructed by exchanging the 5′- and 3′-PCR amplicons generated using specific primer pairs (Bm-M-1: 5′-TATTCGTCTCAGGGAGCAAAAGCAGGTAG-3′, Bm-H6M-711R: 5′-TTTCGTCTCAAATTTTCAA-3′ and Bm-H6M-706: 5′-TTTCGTCTCAAATTTGCAG-3′, Bm-M-1027R: 5′-ATATCGTCTCGTATTAGTAGAAACAAGGTAGTTTTT-3′). Each 7 + 1 reassortant virus was rescued using 293T cells following the method previously described [19]. Briefly, 293T cells were grown to 70% confluency in a 75 cm^2^ flask and then trypsinized with trypsin-EDTA (Life Technologies) and resuspended in 10 mL of Opti-MEM I (Life Technologies). Twenty milliliters of fresh Opti-MEM I was added to 2 mL of cell suspension, and 3 mL of this suspension was seeded into each well of a 6-well tissue culture plate (approximately 10^6^ cells per well). The plates were incubated at 37°C overnight. The following day, 1 *μ*g of each plasmid and 16 *μ*L of TransIT LT-1 (Mirus) transfection reagent were added to Opti-MEM I to a final volume of 200 *μ*L and the mixture was incubated at room temperature for 45 min. After incubation, the medium was removed from one well of the 6-well plate, 800 *μ*L of Opti-MEM I was added to the transfection mix, and this mixture was added dropwise to the cells. Six hours later, the DNA-transfection mixture was replaced by Opti-MEM I. Forty-eight hours after transfection, the cell culture supernatant was inoculated into chicken eggs, and the allantoic fluid was harvested as a seed virus and used the following experiment.

### 2.2. Kinetics of Viral Replication in MDCK Cells and Chickens

Reassortant viruses were titrated using a plaque assay [20]. Plaque size was measured and classified according to diameter. To evaluate the efficiency of virus replication in MDCK cells, viruses at a low multiplicity of infection (MOI) (= 0.001) were used to inoculate semiconfluent cells, and virus titers of culture supernatants harvested every 12 h were determined by plaque assay.

We used 4-week-old white leghorn chickens to evaluate the replication of the reassortants. Infectious allantoic fluid (10^6^ EID_50_) generated using each type of reassortant was intranasally administrated to three chickens each. Birds were examined daily for clinical signs, and tracheal and cloacal swabs were taken three and five days after inoculation. Tracheal and cloacal swabs were placed in 0.5 mL or 1 mL of sample medium [16], respectively. Each sample was titrated on embryonated chicken eggs to determine the virus yield [16].

## 3. Results 

### 3.1. Rescue of 7 + 1 Reassortant Viruses

The reassortant viruses generated using the W312 and ST viral genomes were rescued to determine the viral gene segment required for efficient replication. The supernatant of 293T cell transfected with the 7 + 1 combinations of plasmids was used to inoculate embryonated chicken eggs, and the allantoic fluid was used to determine whether virus was rescued or not by the hemagglutination (HA) test. High titers of eight reassortants with the W312 background and the ST gene segment were rescued; however, reassortants with the ST background and the W312 gene fragment were not detected though blind passage of the allantoic fluid was attempted (data not shown).

### 3.2. Characterization of Plaque Formation and Recovery of Virus from Experimental Infected Chickens

**Figure 1:**
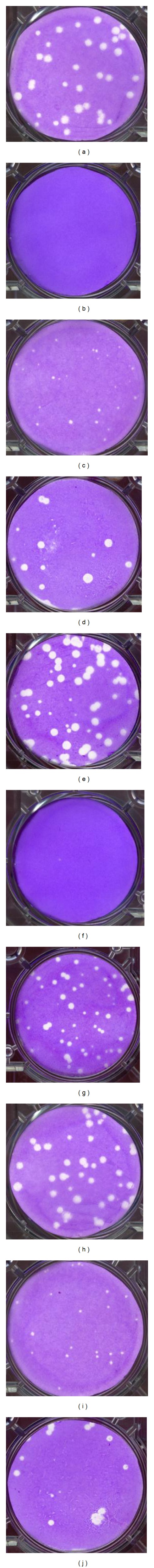
Representative characteristics of plaque formed on MDCK cell cultures. Typical large plaques were induced by the parental W312 strain (a). The plaque size of each reassortant virus with ST-PB1 (d), ST-PA (e), ST-NP (g), ST-NA (h), and ST-NS (j) was similar to that of the W312 strain. The reassortant virus with ST-PB2 (c) and ST-M (i) formed obviously smaller plaque compared with the other reassortants. Countable clear plaques were not detected when parental ST strain (b) and the virus harbored the ST-HA gene (f) resulting in forming the same characteristics in the cells as that of ST strain.

The 7 + 1 reassortants were generated on the background of the parental W312 strain by replacing each genomic fragment with that of the ST strain and were then used to infect MDCK cells. The results of the plaque assays show that the reassortants with ST-PB2 and ST-M genes formed smaller plaques compared with the parental W312 strain or reassortants with other ST-fragments, except for the HA fragment (Figure 1). The virus with the ST-HA fragment or the parental ST strain did not form a countable clear plaque on MDCK cells.

Groups of three chickens intranasally inoculated with 10^6^ EID_50_ of each reassortant did not exhibit detectable clinical symptoms. Viruses with ST-internal gene, except for ST-PB2, ST-HA, and ST-M genes, were recovered from trachea swab samples acquired on days 3 and 5 (Table 1). The reassortants with ST-PB2 or ST-M genes were not recovered from each sample, and the virus with the ST-HA gene was only recovered at low yield from the day 5 sample. No virus was recovered from cloacal sample on both days.

### 3.3. Replication in MDCK Cells and Chickens of Reassortants with Harboring Chimeric PB2 or M Genes

**Figure 2:**
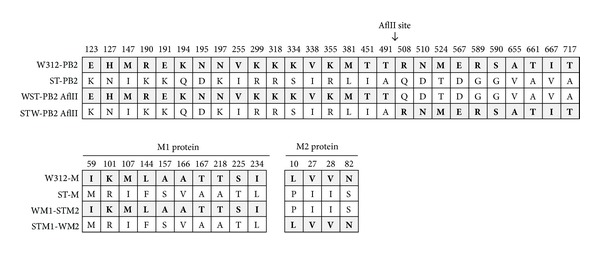
Diagram of the PB2 and M proteins expressed by recombinant viral genomes. Each number indicates the position of amino acid residues that differ between A/teal/Hong Kong/W312/97 (H6N1) and A/duck/Shantou/5540/01 (H6N2) strains. Chimeric PB2 genes were generated by digesting DNAs with *Afl*II, exchanging the fragments, and then ligating them to the pHW2000 vector. To create a chimeric M gene, specifically designed primer sets were used to amplify M1 and M2 sequences, and then the exchanged fragments were ligated to the pHW2000 vector.

**Figure 3:**
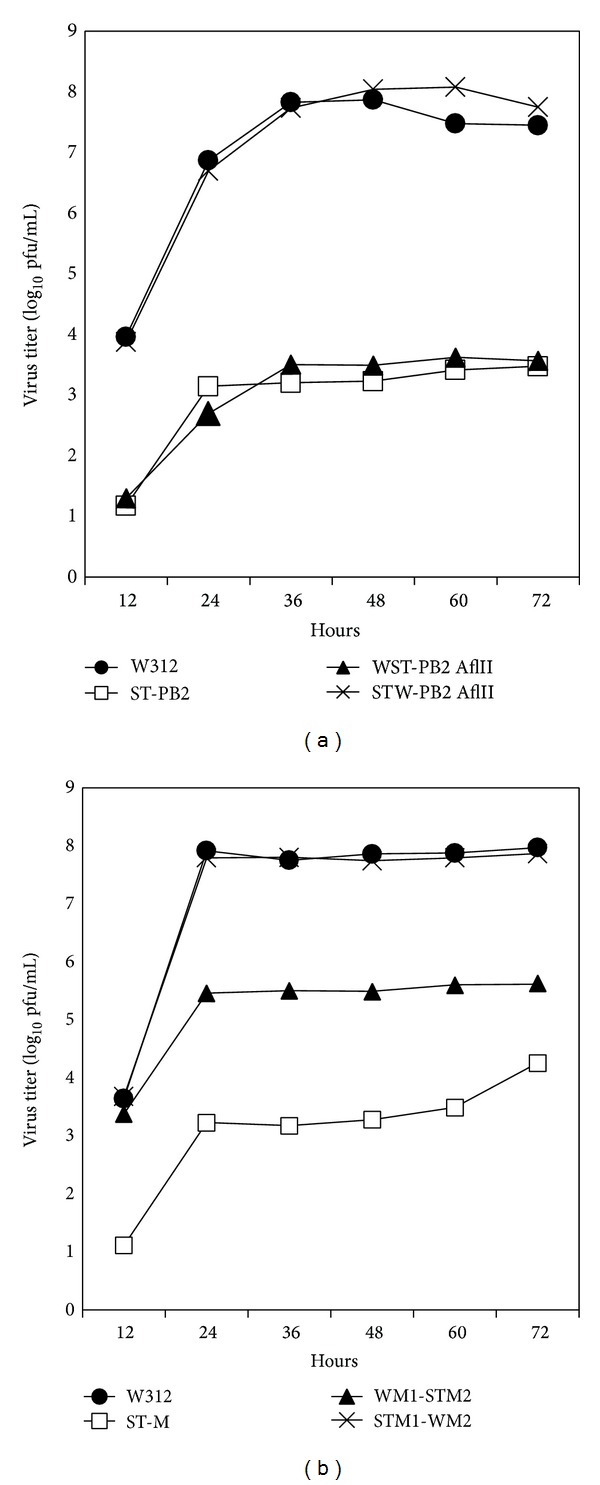
Replication of the parental and reassortant viruses in MDCK cells. MDCK cells were infected with each virus at an MOI = 0.001. Each plot displays average results of three independent experiments. Panel (a) shows the growth kinetics of parental W312 strain versus the virus with ST-PB2 gene or the viruses with chimeric PB2 genes. Panel (b) shows the growth kinetics of parental W312 strain versus the virus with ST-M gene or the viruses with chimeric M genes.

To identify the responsive region on the PB2 and M genes required for effective virus replication, we generated mutant viruses harboring chimeric PB2 or M genes. The deduced amino acid sequences of PB2, M1, and M2 proteins revealed the presence of 27, 10, and 4 amino acid residue substitutions, respectively. The translational products of the genomes generated by recombining the PB2 and M genes are shown in Figure 2. Each reassortant harboring a chimeric gene was evaluated as described above, and their replication kinetics were also determined. The yields of the WST-PB2 *Afl*II mutant propagated in MDCK cells were lower than that produced by the parental strain by a factor of approximately 10^3^. The WST-PB2 *Afl*II virus was not detected in samples taken on days 3 and 5 after inoculation. In contrast, the growth characteristics of STW-PB2 *Afl*II virus both in MDCK and chicken trachea on days 3 and 5 after inoculation were similar to those of W312 strain (Figure 3(a) and Table 1). The virus expressing the STM1-WM2 protein replicated as efficiently as and with yields similar to the parental W312 strain. In contrast, the reassortant virus expressing WM1-STM2 protein replicated differently from the virus with the full-length M gene derived from the ST strain. The yields of the virus expressing the WM1-STM2 protein were approximately lower by a factor of 10^2^ compared with the parental W312 strain and the reassortant virus expressing the STM1-WM2 protein (Figure 3(b)). When chickens were inoculated with these viruses the highly replication-competent STM1-WM2 virus was recovered from the samples on both days; however, the other replicated poorly and was recovered only from the sample taken on day 5. These viruses were not isolated from cloacal samples (Table 1).

## 4. Discussion

The H6N1 influenza virus strain W312 isolated in Hong Kong in 1997 has seven of its eight gene segments in common with those of the A/Hong Kong/156/97 (H5N1) virus that infected humans [13]. In addition, Cheung et al. [11] found that the H6N1/N2 subtype viruses identified in the same region from 2000 to 2005 were derived from W312-like virus, and their genomes may reassort continually with those of H5N1 and H9N2 viruses. Moreover, Huang et al. [21] reported that H6 viruses recently isolated from domestic ducks in southern China represent established multiple lineages, but gene exchange was very limited between the H6N1 lineage in poultry and one gene group in ducks. However, H6 viruses were isolated from chickens in Taiwan, Korea, and California [9, 12, 15, 22]. Among them, H6N1 viruses in Taiwan have circulated in chicken with respiratory distress since 1997 [12]. If the results from Taiwan, Korea, and California indicate that those H6 viruses have been adapted to chicken population, some important mechanisms that confer the ability to adapt and replicate in chickens should exist.

The findings of the present study indicate that genes encoding viral internal proteins are possibly responsible for determining host range. We generated 7 + 1 reassortant viruses with the W312 background that harbored viral genomic sequences derived from the duck strain. The reassortants with reciprocal recombinants between each genome were not rescued even after several attempts. Although this reason was unclear, some viral fragments combinations derived from the duck isolate are potentially related to the difficulty of the viral particle formation in this system.

The structure of the HA molecule influences viral host range, and it is a key factor for infection of domestic chicken population. In particular, H9N2 viruses isolated in southern China, such as the Y280 and G1 lineages, may have adapted to replicate in chickens [23–27]. The results of the present study show that the H6 reassortant with the ST-HA gene was recovered later and at lower titers compared with the parental W312 strain, indicating that the structure of the HA protein acquired from the duck-tropic strain did not support efficient infection of chickens. Although we previously reported that H6 viruses had not completely adapted to chickens around 2003 [16], the W312 strain and viruses in its lineage may have gradually adapted. Lee et al. [12] concluded that the genes encoding the HA and internal proteins of the Taiwanese H6N1 strain isolated from chickens may have mutated to enable it to replicate in domestic chickens.

Moreover, we show here that when chickens were inoculated with reassortants harboring the ST-PB2 and ST-M genes, virus replication in the trachea was repressed. Further, replication was lowest in chicken trachea and cultured cells when the viruses harbored the C-terminal region of the PB2 protein and the M2 protein derived from the W312 strain. Sorrell and Perez [28] described that minor changes in internal genes virus were responsible for the ability of the H2N2 strain, which was isolated from mallards, to replicate in quail and chickens. In particular, the C-terminal region of the PB2 protein has a common amino acid substitution, which differs from that of the mallard-derived virus, allowing it to adapt to chicken. Although the W312 strain was isolated from teals, the viral proteins may have amino acid residue substitutions in common with chicken-adapted H6 viruses such as the Taiwanese isolates. Our results show that the recombinant WM1-STM2 gene conferred intermediate replication level on viruses propagated on cultured cells and chicken trachea. This result indicates that the M1 protein of the W312 strain may be required for efficient virus replication. The findings of Nayak et al. [29] indicate that the M2 protein may play a critical role in the pinching-off process. Thus, when M2 localizes to the neck of the bud, it may facilitate bud release [30] by recruiting nonlipid rafts to this structure. The absence of the M1 protein beneath the lipid bilayers and the absence of spikes on the outer virion surface may indicate the absence of lipid rafts. Such lipid microdomains have been proposed as the preferred sites for the buds to pinch off [31]. Thus, specific host-cell phenotypes may differentially influence virus replication.

Defining the factors that determine host range, particularly those that enable the influenza virus to cross species barriers, is critically important to efforts to prevent the expansion of influenza A virus among mammals. Further analysis of factors that determine host range will be useful for global surveillance to prevent future influenza pandemics.

## Figures and Tables

**Table 1:** Experimental infection of 7 + 1 reassortant H6 viruses.

Virus	No. of positive trachea/total no. of birds [log⁡_10_⁡(EID_50_/mL) for each bird]	
	Day 3	Day 5
Parental strain		
A/teal/Hong Kong/W312/97 (H6N1)	3/3 (3.5, 3.0, 3.7)	3/3 (4.0, 2.5, 3.7)
A/duck/Shantou/5540/01 (H6N2)	0/3 (—, —, —)	0/3 (—, —, —)
Reassortants (W312 backbone)		
ST-PB2	0/3 (—, —, —)	0/3 (—, —, —)
ST-PB1	3/3 (3.7, 4.0, 2.7)	3/3 (3.0, 3.5, 3.0)
ST-PA	3/3 (3.5, 3.5, 3.0)	3/3 (3.5, 3.0, 3.5)
ST-HA	0/3 (—, —, —)	2/3 (—, 1.0, 1.5)
ST-NP	3/3 (2.7, 3.0, 2.7)	3/3 (3.0, 4.0, 3.3)
ST-NA	3/3 (3.5, 3.5, 3.5)	3/3 (3.5, 3.5, 3.0)
ST-M	0/3 (—, —, —)	0/3 (—, —, —)
ST-NS	3/3 (3.3, 3.5, 4.0)	3/3 (3.5, 3.5, 3.7)
Chimeric PB2 gene		
WST-PB2 *Afl*II	0/3 (—, —, —)	0/3 (—, —, —)
STW-PB2 *Afl*II	3/3 (3.5, 3.0, 3.5)	3/3 (3.5, 3.0, 3.5)
Chimeric M gene		
WM1-STM2	0/3 (—, —, —)	1/3 (1.0, —, —)
STM1-WM2	3/3 (2.7, 3.0, 3.7)	3/3 (3.0, 3.0, 3.3)

— Indicates no HA was detected in allantoic fluid from eggs inoculated with original swab sample. Detection limit was 0.5 [log⁡_10_⁡(EID_50_/mL)]. No virus was recovered from cloacal samples on both days.
